# Remyelination varies between and within lesions in multiple sclerosis following bexarotene

**DOI:** 10.1002/acn3.51662

**Published:** 2022-09-17

**Authors:** J. William L. Brown, Ferran Prados, Daniel R. Altmann, Baris Kanber, Jonathan Stutters, Nick G. Cunniffe, Joanne L. Jones, Zoya G. Georgieva, Edward J. Needham, Cyrus Daruwalla, Claudia Gandini Wheeler‐Kingshott, Peter Connick, Siddharthan Chandran, Robin Franklin, David MacManus, Rebecca Samson, Alasdair Coles, Declan Chard

**Affiliations:** ^1^ Department of Clinical Neurosciences University of Cambridge Cambridge UK; ^2^ NMR Research Unit Queen Square Multiple Sclerosis Centre, University College London (UCL) Queen Square Institute of Neurology London UK; ^3^ Clinical Outcomes Research Unit (CORe) University of Melbourne Melbourne Australia; ^4^ e‐Health Center, Universitat Oberta de Catalunya Barcelona Spain; ^5^ Department of Medical Physics and Biomedical Engineering, Centre for Medical Image Computing University College London London UK; ^6^ Medical Statistics Department London School of Hygiene & Tropical Medicine London UK; ^7^ National Institute for Health Research Biomedical Research Centre, University College London Hospitals NHS Foundation Trust and University College London London UK; ^8^ Brain Connectivity Centre, IRCCS Mondino Foundation Pavia Italy; ^9^ Department of Brain and Behavioural Sciences University of Pavia Pavia Italy; ^10^ Centre for Clinical Brain Sciences University of Edinburgh Edinburgh UK; ^11^ UK Dementia Research Institute, University of Edinburgh Edinburgh UK; ^12^ Wellcome‐MRC Cambridge Stem Cell Institute University of Cambridge Cambridge UK

## Abstract

**Objective:**

In multiple sclerosis chronic demyelination is associated with axonal loss, and ultimately contributes to irreversible progressive disability. Enhancing remyelination may slow, or even reverse, disability. We recently trialled bexarotene versus placebo in 49 people with multiple sclerosis. While the primary MRI outcome was negative, there was converging neurophysiological and MRI evidence of efficacy. Multiple factors influence lesion remyelination. In this study we undertook a systematic exploratory analysis to determine whether treatment response – measured by change in magnetisation transfer ratio – is influenced by location (tissue type and proximity to CSF) or the degree of abnormality (using baseline magnetisation transfer ratio and T1 values).

**Methods:**

We examined treatment effects at the whole lesion level, the lesion component level (core, rim and perilesional tissues) and at the individual lesion voxel level.

**Results:**

At the whole lesion level, significant treatment effects were seen in GM but not WM lesions. Voxel‐level analyses detected significant treatment effects in WM lesion voxels with the lowest baseline MTR, and uncovered gradients of treatment effect in both WM and CGM lesional voxels, suggesting that treatment effects were lower near CSF spaces. Finally, larger treatment effects were seen in the outer and surrounding components of GM lesions compared to inner cores.

**Interpretation:**

Remyelination varies markedly within and between lesions. The greater remyelinating effect in GM lesions is congruent with neuropathological observations. For future remyelination trials, whole GM lesion measures require less complex post‐processing compared to WM lesions (which require voxel level analyses) and markedly reduce sample sizes.

## Introduction

In multiple sclerosis, most lesions are thought to go through a phase of inflammatory demyelination followed by a variable degree of remyelination. In lesions that do not fully remyelinate, chronic axonal loss occurs due to loss of trophic support,^1^ and this contributes to irreversible and progressive disability.^2^ A major unmet goal of multiple sclerosis treatment is to enhance endogenous remyelination, so restoring neuronal function and preventing demyelination‐associated chronic axonal loss.

Endogenous remyelination in multiple sclerosis lesions is highly variable, and several factors have been implicated.^3^ Increasing age is a major determinant of remyelination failure.^4^ A substantial proportion of lesions remain chronically active, with evidence of ongoing, if more subtle, inflammation and demyelination at their edges, and such lesions are associated with progressive disability.^5^, ^6^ Lesion location also appears to be relevant; histopathological studies have suggested that lesions in brain grey matter (GM) may remyelinate more effectively than those in white matter (WM), although to the best of our knowledge this has yet to be shown in vivo.^7^, ^8^, ^9^ Within and between WM lesions there may be significant differences: combined PET‐MRI analyses show substantial heterogeneity of demyelination and remyelination in voxels from the same lesion,^10^ and lesions close to the ventricles exhibit greater damage compared with those in deeper tissues,^11^, ^12^, ^13^, ^14^ although it is unknown if this gradient reflects greater damage as lesions form and evolve, or a lower potential to remyelinate.

In remyelination trials, the most frequently employed imaging method for assessing remyelination is measurement of magnetisation transfer ratio (MTR).^15^ In WM and GM, MTR most strongly correlates with myelin density, though in WM there is also a correlation with axonal count and inflammation.^16^, ^17^, ^18^, ^19^ Previous work has shown that assessing remyelination treatment effects in individual rather than pooled lesions increases sensitivity,^20^ and has suggested that this should be restricted to lesions with lower than average MTR.^21^ However, to date, other factors that could influence lesion remyelination have not, to the best of our knowledge, been assessed in vivo.

We have tested the ability of bexarotene, a retinoid X receptor agonist, to promote remyelination in people with relapsing–remitting multiple sclerosis in a phase IIa placebo‐controlled trial (CCMR‐One^22^). The novel MRI efficacy outcome measure – change in MTR in whole lesions whose baseline MTR was below the within‐patient median – was not met, and bexarotene was poorly tolerated, precluding its further development as a treatment in multiple sclerosis. However, statistically significant treatment effects were observed in whole deep grey matter (DGM) lesions, cortical grey matter (CGM) lesions and brainstem lesions, yet not in periventricular, deep‐white matter, juxtacortical nor cerebellar lesions. The interpretation of these MTR changes as representing remyelination is congruent with the observation that bexarotene also reduced the latency of visual evoked potentials (VEPs) in eyes previously affected by optic neuritis.^22^

Here we explore lesional factors that influence the capacity for remyelination using the CCMR‐One trial MRI dataset and calculate necessary sample sizes for future trials of other potential remyelinating agents using the most promising and pragmatic lesion metrics.

## Materials and Methods

### Participants

The Cambridge Centre for Myelin Repair Trial Number One (CCMR‐One, ISRCTN14265371) was a double‐blind phase 2a trial in people with relapsing–remitting multiple sclerosis from two UK centres, aged 18–50 years who had been stable on dimethyl fumarate for at least 6 months. Full inclusion criteria, baseline demographics and methodology can be found in the original trial manuscript.^22^ Briefly, patients were randomised to receive bexarotene or placebo tablets for 6 months and underwent MRI and VEPs at baseline and 6 months. The trial was approved by London Westminster National Research Ethics Service Committee (15/LO/0108) and all participants gave written informed consent at enrolment.

### MRI

#### Acquisitions

One Siemens 3T Prismafit scanner (Siemens, Erlangen, Germany) was used at each site with 20‐channel head–neck coils. Baseline and month 6 scans included the following sequences: 3D magnetisation transfer imaging (for calculation of MTR maps: 1 × 1 × 1 mm, TR = 35 msec, TE = 4.07/9.49 msec, flip angle 9°), 3DT1 (for volumetric measures and segmentation: 1 × 1 × 1 mm, TR = 2400 msec, TE = 2.99 msec, flip angle = 8°), interleaved proton‐density/T2‐weighted scans (for identification and contouring of T2 hyperintense lesions: 1 × 1 × 3 mm, TR = 3050 msec, TE = 31/82 msec) and fluid‐attenuated‐inversion recovery (FLAIR, to assist with lesion identification: 1 × 1 × 3 mm, TR = 9500 msec, TE = 123 msec).

#### Processing

We employed three levels of analysis to explore treatment effects: within whole‐lesions (the most simple to process and therefore the most pragmatic for future remyelination trials); within lesion spatial components (the inner core compared with the outer rim of lesions, and their surrounding normal‐appearing tissues) and lesion segments (regions defined by their baseline MTR and T1‐hypointensity); and within individual lesion voxels (potentially the most sensitive assessment given the aforementioned heterogeneity within lesions, but potentially the most vulnerable to methodological artefacts such as partial volume effects at lesion borders and subtle registration inaccuracies). At a whole‐lesion level, we compared the effect of lesion location (GM compared with WM and distance from cerebrospinal fluid) and the degree of baseline abnormality (based on MTR and T1‐hypointensity). And finally, at a voxel‐level, we compared treatment effects according to location (again GM compared with WM and distance from cerebrospinal fluid) and baseline voxel MTR and T1.

The lesion segmentation and tissue segmentation processing are detailed in the original trial manuscript (and in the Data S1).

To examine spatial components, lesions were segmented into an outer rim (by eroding each T2‐contoured lesion by one voxel‐layer) and a central core (the remaining central tissue). Three concentric perilesional cuffs of normal‐appearing tissue were created by dilating each T2‐contoured lesion by one voxel‐layer (first cuff), then another voxel layer (2nd cuff) and a final voxel layer (3rd cuff). This was done by 3D 1‐voxel dilations. Any voxel included in the cuffs of more than 1 lesion was excluded.

The MTR maps at month 0 and month 6 were calculated directly in the subject halfway volumetric T1 space as follows: (((MToff − MTon)/MToff) × 100). To compute MTR maps in the halfway volumetric T1 space, we concatenated two transformations: one was the transformation between MTon and MToff to the correspondent T1 volumetric scan at each timepoint, and the second was the transformation to the halfway volumetric T1 space. In that way, all scans from month 0 and month 6 were in halfway volumetric T1 space.

Baseline MTR was used for classification in three ways. Firstly, examining MTR treatment effects at the lesion‐level (presented within quartiles of baseline whole‐lesion MTR (for WM lesions) and sub/supramedian (for GM lesions due to insufficient numbers of CGM or DGM lesions for quartiles)); secondly, comparing MTR treatment effects at the lesion segment‐level (where voxels within a lesion were grouped as regions according to their baseline MTR quartile); and thirdly comparing MTR treatment effects at the voxel‐level (presented within quartiles of baseline voxel MTR). Tissue‐specific MTR quartile (or median) values were used for WM lesions, CGM lesions and DGM lesions.

As with baseline MTR, T1 intensity was used for classification in three ways. Firstly, examining MTR treatment effects at the lesion‐level (presented within quartiles of baseline whole‐lesion T1 intensity (for WM lesions) and sub/supramedian (for GM lesions due to insufficient numbers of CGM or DGM lesions for quartiles)); secondly, comparing MTR treatment effects at the lesion segment‐level (where voxels within a lesion were grouped according to their baseline T1 quartile, comparing the mean MTR treatment effect at this lesion‐segment level); and thirdly, comparing MTR treatment effects at the voxel‐level (presented within quartiles of baseline voxel T1 intensity). Tissue‐specific T1 quartile (or median) values were used for WM lesions, CGM lesions and DGM lesions.

### Statistical analyses

With the exception of the sample size calculations, all analyses used linear mixed models with patient and, where appropriate, lesion random intercepts. These models regressed lesion MTR (with lesions nested within patients, for whole lesion analyses), lesion component/segment MTR (with components/segments nested within lesions nested within patients, for component/segment analyses) or individual voxel MTR (with voxels nested within lesions nested within patients, for voxel‐level analyses), adjusting for the baseline value of the outcome measure (lesional MTR, component/segment lesional MTR or voxel MTR respectively) and the four binary minimisation factors prespecified in the trial: age (≤40/> 40 years), gender, trial centre/scanner (Cambridge/Edinburgh) and EDSS (≤4·0/> 4·0 score). We also included lesion‐subgroup interaction terms to (i) estimate lesion‐subgroup specific treatment differences and (ii) test for variation between these differences. The interaction term, which assesses the difference in treatment effects between quartiles or bands, is able to be significant even though the main treatment effect is not, for this reason: these interaction tests are here relatively highly powered because there is a strong within‐patient component, since patients can have lesions in both the quartiles being compared, giving increased precision to the comparison of treatment differences between quartiles; whereas the main treatment difference estimation has to be entirely between‐patient, since no patient can have both treated and untreated lesions. Although analyses at the lesion, component/segment or voxel level are more flexible and powerful than patient‐level analyses, they are vulnerable to selection bias since patients, not lesions or components/segments or voxels, were randomised (and some patients may not have contributed to all analyses): all analyses are therefore exploratory. We repeated each analysis in pure WM lesions, CGM lesions and DGM lesions (each using a tissue‐specific cohort‐level quartile (or median) values). In pure WM lesions, we mitigated partial volume effects by excluding voxels from the outermost layer for voxel‐level and segment‐level analyses. Excluding the outermost layer in CGM and DGM lesions left insufficient numbers (34 CGM lesions and 11 DGM lesions) so was not performed. Low numbers of DGM lesions (*n* = 16) precluded analysing the effect of distance from the ventricles or T1‐hypointensity.

### Sample size calculations

Complete remyelination could not increase lesional MTR beyond that of the NAWM. We previously found that the difference between mean lesional MTR and mean normal‐appearing white matter MTR is 5.92 percentage units (pu),^20^ though ex‐vivo data point to a 50% remyelination ceiling effect, suggesting a maximum treatment effect of 2.96 pu. Necessary sample sizes, for 5% significance and both 80% and 90% power, will therefore be presented to detect five biologically significant treatment effects: 1.3 pu (43% of the maximum), 1.4 pu (47% of the maximum), 1.5 pu (50% of the maximum), 1.6 pu (53% of the maximum), 1.7 pu (57% of the maximum) and 1.8 pu (60% of the maximum).

Sample sizes were standardly calculated for patient‐averaged lesional measures, since patients and not lesions are randomised (and clinical measures are patient‐level); calculations were for an ANCOVA analysis, which is a comparison of active versus placebo means using a *t*‐test but adjusted for baseline, and can be estimated by incorporating the baseline‐follow‐up correlations. This used the placebo group means and standard deviations at follow‐up, and the placebo group baseline vs follow‐up Pearson correlation coefficient. Calculations were performed in Stata (version 16.1, Stata Corporation, College Station, TX, USA).

### Correlation between voxel intensity between baseline and follow‐up

To explore whether the same voxel was being captured at baseline and follow‐up we performed Pearson correlation between the baseline and follow‐up voxel MTR for lesional voxels in patients receiving placebo.

Analyses were performed in Stata version 16.1 and R version 4.1. Results were considered statistically significant at the *p* < 0.05 level.

### Data Availability Statement

The data that support the findings of this study are available from the corresponding author, upon reasonable request.

## Results

All MRI scans from the 49 patients with baseline and follow‐up imaging were of sufficient quality for inclusion, generating 3170 T2 hyperintense lesions (1613 lesions containing only WM (pure WM lesions), 106 lesions containing only GM (pure GM lesions) and 1451 mixed GM and WM lesions). Within the 106 pure GM lesions were 85 CGM lesions, 16 DGM and 5 cerebellar lesions. Due to insufficient numbers, cerebellar lesions were removed from all analyses of pure GM lesions (though were included in sensitivity analyses). Results are presented at the whole‐lesion level (Table 1), the lesion component/segment‐level (Table 2) and the lesional voxel‐level (Table 3).

**Table 1:** MRI outcomes for whole lesion analyses

Subgroup of lesions	Bexarotene		Placebo		Bexarotene‐placebo change	
	Patient number	Unadjusted mean (SD) change in lesional MTR (pu)	Patient number	Unadjusted mean (SD) change in lesional MTR (pu)	Adjusted bexarotene‐placebo difference (95% CI)	*p*‐value
Primary efficacy endpoint (patient‐level), as reported previously^22^					
Patient submedian lesions	25	0.25 (0.98)	24	0.09 (0.84)	0.16 (−0.39, 0.71)	0.554

Exploratory MRI analyses (lesion‐level)	Bexarotene		Placebo		Bexarotene‐placebo change	
	Lesion number	Unadjusted mean (SD) change in lesional MTR (pu)	Lesion number	Unadjusted mean (SD) change in lesional MTR (pu)	Adjusted bexarotene‐placebo difference (95% CI)	*p*‐value
All lesions	1946	0.00 (1.94)	1224	−0.12 (1.61)	0.12 (−0.35, 0.58)	0.629
All pure WM lesions	1003	−0.04 (1.8)	610	−0.08 (1.48)	0.10 (−0.38, 0.68)	0.568
All pure GM lesions	53	0.67 (2.58)	48	−0.6 (2.95)	1.08 (0.32, 1.84)	0.008
All pure CGM lesions	46	0.69 (2.58)	39	−0.42 (3.20)	1.00 (0.12, 1.75)	0.023
All pure DGM lesions	7	0.49 (2.81)	9	−1.41 (1.25)	1.93 (0.28, 3.59)	0.027
Effect of baseline whole lesion MTR						
Quartile 1 pure WM lesions [lowest]	237	0.65 (2.13)	167	−0.02 (1.77)	0.43 (−0.09, 0.96)	0.114
Quartile 2 pure WM lesions	250	0.09 (1.57)	153	0.21 (1.25)	0.22 (−0.30, 0.74)	0.416
Quartile 3 pure WM lesions	246	−0.11 (1.40)	157	−0.01 (1.26)	0.04 (−0.48, 0.55)	0.889
Quartile 4 pure WM lesions [highest]	270	−0.70 (1.77)	133	−0.57 (1.45)	−0.19 (−0.70, 0.33)	0.484
*Interaction test comparing treatment group differences between the quartiles*						0.004
Submedian pure CGM lesions	24	1.55 (2.36)	18	0.31 (2.71)	1.21 (−0.87, 3.28)	0.266
Supramedian pure CGM lesions	22	−0.24 (2.54)	21	−1.04 (3.5)	1.14 (−0.67, 2.95)	0.229
*Interaction test comparing treatment group differences (submedian vs supramedian lesions)*						0.961
Submedian pure DGM lesions	3	1.59 (3.71)	5	−1.16 (1.48)	2.59 (−0.94, 6.11)	0.201
Supramedian pure DGM lesions	4	−0.34 (2.12)	4	−1.73 (1.01)	2.32 (−1.47, 6.11)	0.275
*Interaction test comparing treatment group differences (submedian vs supramedian lesions)*						0.924
Effect of whole baseline lesion size						
Quartile 1 pure WM lesions [lowest]	291	−0.02 (2.04)	170	0 (1.57)	0.05 (−0.457, 0.561)	0.843
Quartile 2 pure WM lesions	209	−0.14 (1.85)	138	−0.11 (1.84)	0.07 (−0.445, 0.589)	0.785
Quartile 3 pure WM lesions	250	0.03 (1.65)	152	−0.19 (1.28)	0.15 (−0.361, 0.666)	0.563
Quartile 4 pure WM lesions [highest]	253	−0.05 (1.58)	150	−0.03 (1.14)	0.13 (−0.381, 0.645)	0.617
*Interaction test comparing treatment group differences between the quartiles*						0.584
Submedian pure CGM lesions	21	1.05 (2.43)	19	0.04 (2.61)	1.86 (−0.08, 3.79)	0.073
Supramedian pure CGM lesions	25	0.39 (2.72)	20	−0.85 (3.68)	0.71 (−1.14, 2.56)	0.458
*Interaction test comparing treatment group differences (submedian vs supramedian lesions)*						0.342
Submedian pure DGM lesions	5	1.17 (3.08)	3	−1.98 (1.52)	2.68 (0.10, 5.26)	0.076
Supramedian pure DGM lesions	2	−1.22 (1.13)	6	−1.13 (1.14)	−0.06 (−3.09, 2.98)	0.971
*Interaction test comparing treatment group differences (submedian vs supramedian lesions)*						N/A
Effect of baseline whole lesion T1						
Quartile 1 pure WM lesions [lowest]	248	−0.45 (1.77)	156	−0.50 (1.40)	0.15 (−0.53, 0.83)	0.661
Quartile 2 pure WM lesions	228	0.31 (1.84)	175	0.30 (1.29)	−0.04 (−0.67, 0.58)	0.895
Quartile 3 pure WM lesions	269	−0.05 (1.98)	134	−0.26 (1.18)	−0.04 (−0.62, 0.54)	0.886
Quartile 4 pure WM lesions [highest]	258	0.06 (1.49)	145	0.09 (1.84)	0.08 (−0.49, 0.65)	0.789
*Interaction test comparing treatment group differences between the quartiles*						0.865
Submedian pure CGM lesions	24	1.34 (2.25)	19	0.00 (2.96)	1.43 (−0.60, 3.47)	0.181
Supramedian pure CGM lesions	22	−0.01 (2.78)	20	−0.81 (3.43)	1.00 (−0.86, 2.86)	0.301
*Interaction test comparing treatment group differences (submedian vs supramedian lesions)*						0.744
Submedian pure DGM lesions	3	1.19 (4.30)	5	−1.76 (1.15)	2.41 (−1.22, 6.04)	0.241
Supramedian pure DGM lesions	4	−0.04 (1.60)	4	−0.98 (1.40)	1.81 (−3.50, 7.13)	0.528
*Interaction test comparing treatment group differences (submedian vs supramedian lesions)*						0.878
Effect of band number						
Pure WM lesions in band 1 (adjacent to the ventricle)	24	−0.85 (1.64)	16	−0.13 (1.32)	−0.03 (−0.71, 0.64)	0.929
Pure WM lesions in band 2	35	0.31 (1.53)	29	0.08 (1.19)	−0.06 (−0.71, 0.59)	0.858
Pure WM lesions in band 3	62	−0.45 (1.73)	34	−0.23 (1.34)	0.02 (−0.61, 0.65)	0.957
Pure WM lesions in band 4	78	−0.42 (1.76)	74	−0.24 (1.28)	−0.02 (−0.63, 0.59)	0.947
Pure WM lesions in band 5	149	0.04 (1.77)	76	0.04 (1.35)	−0.01 (−0.58, 0.56)	0.969
Pure WM lesions in band 6	143	−0.06 (1.57)	85	−0.11 (1.65)	0.08 (−0.47, 0.63)	0.768
Pure WM lesions in band 7	163	0.01 (1.75)	105	−0.03 (1.3)	−0.03 (−0.57, 0.52)	0.927
Pure WM lesions in band 8	192	−0.03 (2.04)	86	0.06 (1.37)	0.07 (−0.47, 0.60)	0.802
*Interaction test comparing treatment group differences between the bands*						0.709
Pure CGM lesions in band 1 (inner)	23	1.1 (2.8)	17	−0.8 (3.72)	2.24 (−1.95, 6.43)	0.305
Pure CGM lesions in band 2 (outer)	23	0.29 (2.33)	22	−0.12 (2.78)	1.52 (−0.45, 3.49)	0.144
*Interaction test comparing treatment group differences between the bands*						0.588

*p* values and 95% CIs are for the differences between bexarotene and placebo after adjustment (for the baseline value of that measure and the four binary minimisation factors).

MTR, magnetisation transfer ratio; NA, not applicable.

**Table 2:** MRI outcomes for lesion component/segment‐level analyses.

	Bexarotene		Placebo		Bexarotene‐placebo change	
	Lesion number	Unadjusted mean (SD) change in component/segment lesional MTR (pu)	Lesion number	Unadjusted mean (SD) change in component/segment lesional MTR (pu)	Adjusted bexarotene‐placebo difference (95% CI)	*p*‐value
Lesion components (defined spatially)						
Core (innermost) pure WM lesions	695	−0.04 (2.17)	414	−0.09 (1.71)	0.07 (−0.42, 0.56)	0.773
Rim pure WM lesions	1003	0.01 (1.79)	610	0 (1.48)	0.11 (−0.38, 0.59)	0.665
Cuff 1 pure WM lesions	997	−0.02 (1.6)	608	−0.06 (1.33)	0.14 (−0.34, 0.63)	0.566
Cuff 2 pure WM lesions	1002	−0.04 (1.5)	610	−0.07 (1.27)	0.13 (−0.36, 0.61)	0.613
Cuff 3 (outermost) pure WM lesions	1002	−0.02 (1.45)	610	−0.06 (1.22)	0.13 (−0.36, 0.61)	0.602
*Interaction test comparing treatment group differences between the components*						0.807
Core (innermost) pure CGM lesions	20	0.27 (4.58)	14	0.16 (2.82)	0.01 (−1.41, 1.43)	0.993
Rim pure CGM lesions	46	1.12 (2.69)	39	−0.23 (3.19)	1.27 (0.05, 2.48)	0.053
Cuff 1 pure CGM lesions	46	0.77 (2.37)	39	−0.69 (2.38)	1.37 (0.15, 2.58)	0.038
Cuff 2 pure CGM lesions	46	0.83 (2.51)	39	−0.25 (1.91)	0.98 (−0.23, 2.20)	0.127
Cuff 3 (outermost) pure CGM lesions	46	0.63 (2.55)	39	0 (1.72)	0.57 (−0.65, 1.78)	0.369
*Interaction test comparing treatment group differences between the components*						0.042
Core (innermost) pure DGM lesions	5	−0.64 (1.65)	6	−1.93 (1.82)	2.09 (0.20, 3.99)	0.063
Rim pure DGM lesions	7	0.47 (2.82)	9	−1.63 (1.42)	2.37 (0.63, 4.10)	0.029
Cuff 1 pure DGM lesions	7	0.28 (0.18)	9	−1.5 (1.44)	2.49 (0.74, 4.23)	0.023
Cuff 2 pure DGM lesions	7	0.18 (2.15)	9	−0.88 (1.95)	1.92 (0.17, 3.68)	0.064
Cuff 3 (outermost) pure DGM lesions	7	0.14 (2.06)	9	−0.65 (1.87)	1.63 (−0.13, 3.38)	0.107
*Interaction test comparing treatment group differences between the components*						0.732
Lesion segments (defined by the baseline MTR of each voxel)						
Quartile 1 (MTR‐defined) segment of pure WM lesions	121	0.99 (2.65)	69	0.52 (1.7)	0.62 (−0.06, 1.31)	0.080
Quartile 2 (MTR‐defined) segment of pure WM lesions	287	0.46 (2.55)	163	0.26 (1.74)	0.23 (−0.37, 0.83)	0.462
Quartile 3 (MTR‐defined) segment of pure WM lesions	431	0.09 (2.13)	265	0.10 (1.72)	0.08 (−0.49, 0.66)	0.776
Quartile 4 (MTR‐defined) segment of pure WM lesions	503	−0.43 (1.93)	306	−0.45 (1.74)	0.14 (−0.42, 0.71)	0.622
*Interaction test comparing treatment group differences between the segments*						0.157
Quartile 1 (MTR‐defined) segment of pure CGM lesions	32	1.61 (3.56)	29	1.39 (3.21)	0.98 (−0.63, 2.58)	0.245
Quartile 2 (MTR‐defined) segment of pure CGM lesions	40	1.27 (3.18)	32	−0.07 (2.64)	1.51 (−0.04, 3.06)	0.070
Quartile 3 (MTR‐defined) segment of pure CGM lesions	42	0.44 (2.60)	35	−0.69 (3.01)	1.40 (−0.13, 2.93)	0.086
Quartile 4 (MTR‐defined) segment of pure CGM lesions	33	−0.26 (2.81)	27	−1.29 (3.40)	1.02 (−0.59, 2.62)	0.227
*Interaction test comparing treatment group differences between the segments*						0.791
Quartile 1 (MTR‐defined) segment of pure DGM lesions	5	1.83 (2.26)	8	−0.96 (2.07)	2.66 (0.60, 4.72)	0.050
Quartile 2 (MTR‐defined) segment of pure DGM lesions	6	0.22 (1.99)	8	−1.15 (1.83)	2.09 (−0.01, 4.18)	0.087
Quartile 3 (MTR‐defined) segment of pure DGM lesions	6	−0.60 (1.60)	8	−1.40 (1.29)	1.49 (−0.61, 3.59)	0.202
Quartile 4 (MTR‐defined) segment of pure DGM lesions	5	−1.19 (1.91)	7	−2.00 (1.76)	2.24 (0.12, 4.37)	0.072
*Interaction test comparing treatment group differences between the segments*						0.489
Lesion segments (defined by the baseline T1 value of each voxel)						
Quartile 1 (T1‐defined) segment of pure WM lesions	108	−0.51 (2.32)	79	−0.32 (1.59)	0.27 (−0.56, 1.11)	0.525
Quartile 2 (T1‐defined) segment of pure WM lesions	197	0.08 (2.04)	191	0 (1.72)	0.09 (−0.62, 0.79)	0.806
Quartile 3 (T1‐defined) segment of pure WM lesions	316	0.04 (2.29)	121	−0.05 (1.31)	−0.02 (−0.69, 0.65)	0.957
Quartile 4 (T1‐defined) segment of pure WM lesions	261	0.08 (2.08)	135	−0.11 (2.13)	0.35 (−0.32, 1.02)	0.309
*Interaction test comparing treatment group differences between the segments*						0.447
Quartile 1 (T1‐defined) segment of pure CGM lesions	20	0.56 (4.49)	17	0.46 (2.51)	0.57 (−1.64, 2.79)	0.616
Quartile 2 (T1‐defined) segment of pure CGM lesions	31	0.09 (2.83)	23	0.21 (3.63)	1.29 (−0.78, 3.36)	0.234
Quartile 3 (T1‐defined) segment of pure CGM lesions	36	0.19 (2.72)	28	−0.91 (3.83)	1.66 (−0.35, 3.66)	0.121
Quartile 4 (T1‐defined) segment of pure CGM lesions	26	0.17 (2.54)	24	−0.25 (3.51)	2.01 (−0.07, 4.08)	0.072
*Interaction test comparing treatment group differences between the segments*						0.544
Quartile 1 (T1‐defined) segment of pure DGM lesions	2	−0.41 (4.01)	4	−2.71 (1.3)	2.90 (−0.23, 6.03)	0.107
Quartile 2 (T1‐defined) segment of pure DGM lesions	4	1 (3.5)	7	−0.99 (1.7)	2.17 (−0.49, 4.84)	0.149
Quartile 3 (T1‐defined) segment of pure DGM lesions	5	0.34 (2.04)	4	0.29 (2.23)	1.19 (−1.62, 4.00)	0.430
Quartile 4 (T1‐defined) segment of pure DGM lesions	3	0.16 (2.42)	2	−1.04 (2.02)	3.00 (−0.50, 6.51)	0.131
*Interaction test comparing treatment group differences between the segments*						0.545

*p* values and 95% CIs are for the differences between bexarotene and placebo after adjustment (for the baseline value of that measure and the four binary minimisation factors). MTR, magnetisation transfer ratio; NA, not applicable.

**Table 3:** MRI outcomes for lesion voxel‐level analyses.

	Bexarotene		Placebo		Bexarotene‐placebo change	
	Voxel number	Unadjusted mean (SD) change in lesional voxel MTR (pu)	Voxel number	Unadjusted mean (SD) change in lesional voxel MTR (pu)	Adjusted bexarotene‐placebo difference (95% CI)	*p*‐value
All voxels	10,921	−0.04 (2.68)	8,384	−0.1 (2.32)	0.32 (−0.23, 0.87)	0.227
All voxels from pure WM lesions	9,650	−0.08 (2.48)	7,125	0.06 (1.93)	0.21 (−0.33, 0.75)	0.445
All voxels from pure GM lesions	1,271	0.26 (3.85)	1,259	−0.98 (3.72)	1.45 (0.16, 2.74)	0.036
All voxels from pure CGM lesions	1,040	0.48 (4.06)	929	−0.85 (4.12)	1.25 (−0.20, 2.70)	0.105
All voxels from pure DGM lesions	231	−0.74 (2.48)	330	−1.33 (2.2)	2.24 (0.44, 4.04)	0.041
Effect of baseline voxel MTR: voxels from pure WM lesions						
Quartile 1 [lowest]	2,225	0.41 (2.71)	1,968	0.53 (2.01)	0.69 (0.13, 1.25)	0.020
Quartile 2	2,199	0.15 (2.53)	1,995	0.02 (1.89)	0.39 (−0.16, 0.94)	0.175
Quartile 3	2,553	−0.15 (2.30)	1,640	−0.01 (1.82)	0.15 (−0.39, 0.70)	0.592
Quartile 4 [highest]	2,673	−0.61 (2.28)	1,521	−0.44 (1.82)	0.04 (−0.50, 0.59)	0.871
*Interaction test comparing treatment group differences between the quartiles*						<0.0001
Effect of baseline voxel MTR: voxels from pure CGM lesions						
Quartile 1 [lowest]	283	1.18 (4.61)	209	0.48 (3.66)	0.81 (−0.71, 2.34)	0.306
Quartile 2	266	1.16 (3.93)	226	−0.40 (3.86)	1.06 (−0.44, 2.56)	0.181
Quartile 3	251	0.50 (3.4)	241	−0.77 (3.54)	1.26 (−0.23, 2.74)	0.112
Quartile 4 [highest]	239	−1.09 (3.68)	253	−2.43 (4.69)	1.62 (0.11, 3.13)	0.047
*Interaction test comparing treatment group differences between the quartiles*						0.047
Effect of baseline voxel MTR: voxels from pure DGM lesions						
Quartile 1 [lowest]	50	0.60 (2.85)	90	−0.87 (2.46)	2.19 (0.36, 4.02)	0.046
Quartile 2	47	−0.54 (2.03)	93	−0.76 (2.19)	2.07 (0.25, 3.89)	0.057
Quartile 3	65	−1.16 (1.92)	75	−1.27 (1.61)	1.75 (−0.05, 3.56)	0.094
Quartile 4 [highest]	68	−1.61 (2.19)	72	−2.68 (1.83)	2.03 (0.17, 3.88)	0.064
*Interaction test comparing treatment group differences between the quartiles*						0.517
Effect of baseline voxel T1: voxels from pure WM lesions						
Quartile 1 [lowest]	2,264	−0.79 (2.58)	1,929	0.3 (1.7)	0.16 (−0.45, 0.78)	0.611
Quartile 2	1,783	0.21 (2.18)	2,411	0.06 (1.96)	−0.02 (−0.60, 0.56)	0.952
Quartile 3	2,810	0.18 (2.62)	1,383	0.18 (2.07)	0.02 (−0.54 0.58)	0.951
Quartile 4 [highest]	2,792	0.05 (2.31)	1,402	−0.42 (1.95)	0.30 (−0.26, 0.85)	0.298
*Interaction test comparing treatment group differences between the quartiles*						0.643
Effect of baseline voxel T1: voxels from pure CGM lesions						
Quartile 1 [lowest]	231	1.81 (4.58)	261	−0.86 (4.69)	1.08 (−0.74, 2.90)	0.258
Quartile 2	348	0.34 (4.05)	144	−0.63 (3.74)	1.44 (−0.26, 3.15)	0.112
Quartile 3	259	−0.08 (3.79)	233	−1.21 (3.77)	1.49 (−0.04, 3.03)	0.070
Quartile 4 [highest]	201	−0.11 (3.35)	291	−0.66 (4.01)	1.50 (−0.06, 3.07)	0.074
*Interaction test comparing treatment group differences between the quartiles*						0.104
Effect of baseline voxel T1: voxels from pure DGM lesions						
Quartile 1 [lowest]	45	−2.36 (2.88)	95	−2.07 (1.79)	2.35 (0.33, 4.38)	0.052
Quartile 2	40	1.49 (2.78)	100	−0.72 (2.38)	2.04 (0.11, 3.97)	0.072
Quartile 3	71	−0.2 (1.41)	69	−0.25 (2.26)	1.77 (−0.23, 3.78)	0.121
Quartile 4 [highest]	74	−1.45 (1.8)	66	−2.29 (1.51)	2.15 (0.17, 4.13)	0.066
*Interaction test comparing treatment group differences between the quartiles*						0.711
Effect of voxel band number: voxels from pure WM lesions						
Band 1 [nearest ventricles]	184	−0.15 (1.69)	242	−0.03 (1.72)	−0.15 (−0.73, 0.43)	0.612
Band 2	451	0.13 (2.25)	596	0.24 (1.82)	−0.10 (−0.67, 0.47)	0.727
Band 3	732	−0.32 (2.35)	760	−0.07 (1.84)	−0.05 (−0.61, 0.51)	0.858
Band 4	1,067	−0.44 (2.33)	930	−0.29 (1.73)	0.05 (−0.50, 0.60)	0.857
Band 5	1,729	−0.30 (2.33)	1,051	−0.18 (1.76)	0.24 (−0.31, 0.79)	0.389
Band 6	1,796	−0.05 (2.45)	1,101	0.04 (1.90)	0.17 (−0.38, 0.72)	0.552
Band 7	1,649	0.03 (2.44)	1,087	0.09 (1.98)	0.18 (−0.36, 0.73)	0.515
Band 8	1,393	0.17 (2.86)	838	0.25 (2.16)	0.09 (−0.46, 0.64)	0.743
*Interaction test comparing treatment group differences between the bands*						0.004
Effect of voxel band number: voxels from pure CGM lesions						
Band 1 (inner)	512	1.02 (4.04)	434	−0.87 (4.05)	2.97 (1.25, 4.70)	0.003
Band 2 (outer)	528	−0.04 (4.00)	495	−0.83 (4.18)	1.84 (0.36, 3.31)	0.024
*Interaction test comparing treatment group differences between the bands*						0.0003

*p* values and 95% CIs are for the differences between bexarotene and placebo after adjustment (for the baseline value of that measure and the four binary minimisation factors). MTR, magnetisation transfer ratio; NA, not applicable.

### Lesion location

We have previously reported the significant treatment effects seen in whole DGM, CGM and brainstem lesions.^22^

### Grey matter versus white matter

Significant treatment effects were seen in pure whole GM lesions (adjusted bexarotene‐placebo difference 1.08 pu, 95% CI 0.32–1.84, *p* = 0.008) but not in pure whole WM lesions (adjusted bexarotene‐placebo difference 0.10 pu, 95% CI ‐0.38 to 0.68; *p* = 0.57). This remained so when the 5 pure GM cerebellar lesions were included in the pure GM lesions group (adjusted bexarotene‐placebo difference 1.07 pu, 95% CI 0.32 to 1.83, *p* = 0.007). Significant treatment effects were not seen in individual voxels from pure WM lesions (adjusted bexarotene‐placebo difference 0.21 pu, 95% CI −0.33 to 0.75, *p* = 0.445), but again were seen in voxels from pure GM lesions (adjusted bexarotene‐placebo difference 1.45 pu, 95% CI 0.16 to 2.74, *p* = 0.037).

### Proximity to brain surface

Lesions were classified depending on which concentric band they were mainly located in (as an indicator of proximity to the brain surface, see Fig. S1). In pure WM lesions, band number had no detectable influence on treatment effects (interaction term *p* = 0.709, Table 1). When individual voxels from pure WM lesions were classified by band, no statistically significant treatment effects were seen in any band, but the treatment effects were significantly different between the bands (interaction term *p* = 0.004), increasing with distance from the CSF from bands 1 to 5 (Table 3). There were also no significant treatment effects seen when whole pure CGM lesions were classified based on their predominant cortical band (and the treatment effects were not significantly different between bands (interaction term *p* = 0.588)). However, individual voxels from these same CGM lesions showed statistically significant treatment effects in both bands, and the treatment effect was significantly greater in the innermost band (furthest from the CSF, interaction term *p* = 0.0003). Low lesion numbers precluded exploring the relationship between treatment effect and band number in DGM lesions or their voxels.

### Baseline features

#### Baseline MTR (whole‐lesion level, MTR‐defined segment‐level and voxel‐level analyses)

**Figure 1:**
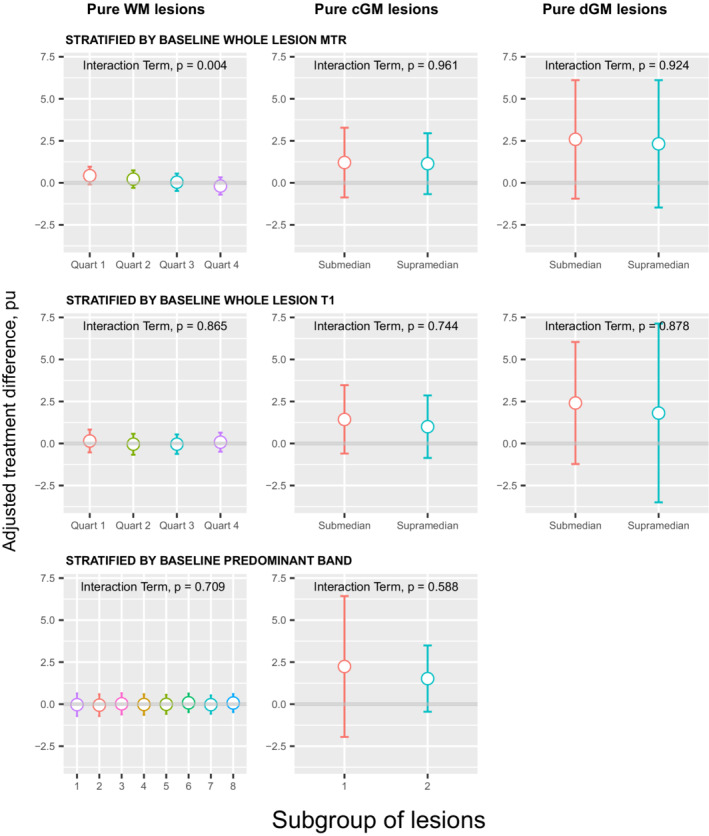
Adjusted treatment difference of whole lesion MTR (pu) in pure WM lesions, pure CGM lesions and pure DGM lesions. Each row has stratified the lesions according to a different baseline feature. Increasing treatment difference favours bexarotene. Error bars represent 95% confidence intervals. Interaction term examines differences in treatment effects between subgroups of lesions.

The tissue‐specific baseline whole‐lesion MTR was used to categorise lesions into quartiles (for pure WM lesions) or into submedian and supramedian categories (for CGM and DGM lesions, which had insufficient numbers for quartiles). Within whole pure WM, CGM and DGM lesions, no significant treatment effects were seen in any category of baseline MTR. However, in pure WM lesions, a significant difference was seen between the treatment effects in each quartile of baseline MTR (interaction term *p* = 0.004) indicating an increasing effect with decreasing baseline MTR (Table 1; Fig. 1).

When the same lesions were segmented based on the baseline MTR quartile of each voxel, treatment effects in all segments did not reach statistical significance, and treatment effects were not significantly different between segments within pure WM, CGM or DGM lesions (Table 2).

**Figure 2:**
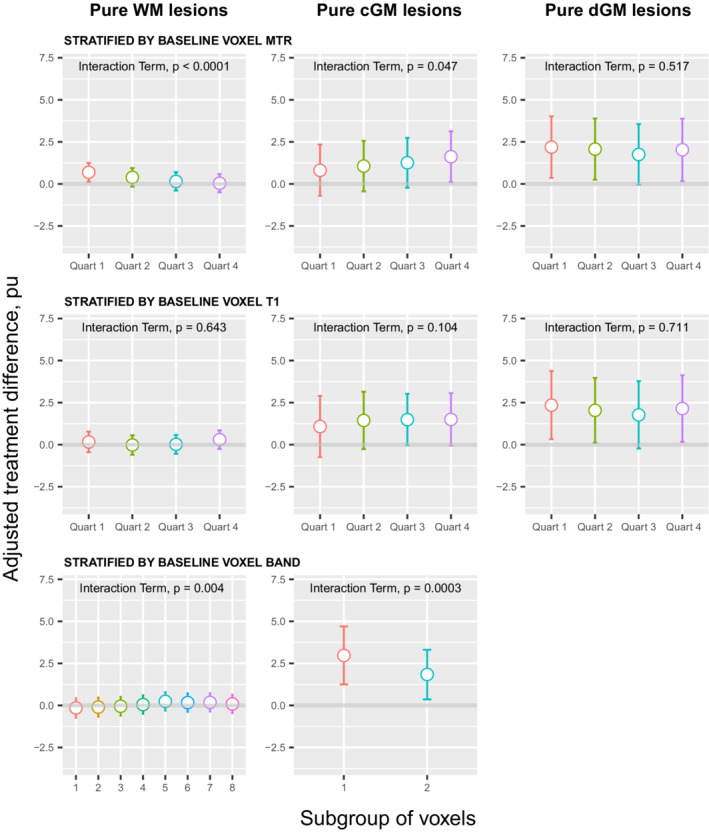
Adjusted treatment difference of voxel‐level lesional MTR (pu) within pure WM lesions, pure CGM lesions and pure DGM lesions. Each row has stratified the voxels according to a different baseline features. Increasing treatment difference favours bexarotene. Error bars represent 95% confidence intervals. Interaction term examines differences in treatment effects between subgroups of voxels.

When individual voxels from the same pure WM lesions were stratified into baseline MTR quartiles, treatment effects increased as the MTR quartile decreased (interaction term *p* < 0.0001) with a statistically significant treatment effect observed in the lowest quartile (Table 3; Fig. 2). Conversely, in voxels from CGM lesions, treatment effects decreased as the MTR quartile decreased (*p* = 0.047) with a statistically significant treatment effect observed in the highest quartile of pure CGM lesions. The treatment effect in the small number of voxels from DGM lesions did not significantly vary between quartiles.

To ensure that lesion‐level, lesion segment‐level and lesion voxel‐level analyses examined the same tissue (to enable comparison of the methods), the whole‐lesion analysis was repeated after excluding the outermost voxels layers in pure WM lesions. No significant treatment effects were seen in any quartile of baseline MTR (quartile 1: adjusted bexarotene‐placebo difference (95% CI) 0.32 (−0.32 to 0.96) pu, *p* = 0.331; quartile 2: 0.16 (−0.47 to 0.79) pu, *p* = 0.630; quartile 3: 0.34 (−0.29 to 0.96) pu, *p* = 0.298; quartile 4–0.13 (0.76 to 0.51) pu, *p* = 0.689) with no significant difference between the treatment groups (interaction term *p* = 0.384).

#### Baseline T1 (whole‐lesion level, T1‐defined segment‐level and voxel‐level analyses)

The tissue‐specific baseline whole‐lesion T1 was used to categorise lesions into quartiles (for pure WM lesions) or into submedian and supramedian categories (for CGM and DGM lesions, which had insufficient numbers for quartiles). Within whole pure WM, CGM and DGM lesions, no significant treatment effects were seen in any category of baseline T1 (Table 1; Fig. 1), and no significant difference was seen between categories.

When the same lesions were segmented based on the T1 quartile of each voxel, treatment effects in all segments did not reach statistical significance, and treatment effects were not significantly different between segments within pure WM, CGM or DGM lesions (Table 2).

When individual voxels were stratified into quartiles based on their baseline T1 value, no statistically significant MTR treatment effects were seen in any quartile (pure WM, CGM or DGM lesions) and the treatment effects were not significantly different between any quartile (Table 3; Fig. 2).

#### Baseline size

No statistically significant treatment effect was seen in lesions based on their size in any tissue type (Table 1).

### Spatial lesion‐components

In pure WM lesions there was no difference in treatment effects between the inner core and outer rim (Table 2). In CGM and in DGM the outer rim and first surrounding normal‐appearing GM cuffs showed statistically significant treatment effects while the cores did not.

### Correlation between voxel intensity between baseline and follow‐up

The correlation coefficients between baseline and follow‐up lesional voxel MTR across all lesion subtypes were strong (Pearson correlation coefficients 0.737 to 0.955, see Table S1).

### Sample size calculations

Calculated sample sizes using the most promising whole‐lesion metrics are presented in Table 4, alongside their respective baseline‐adjusted treatment effects observed during the CCMR‐One trial (range 0.923–2.000 pu). For comparison, the total sample size necessary to detect the observed baseline‐adjusted treatment effect averaged over all of a patient's lesions (0.144 pu) is 882 people (at 80% power) or 1180 people (at 90% power); and to detect the observed baseline‐adjusted treatment effect averaged over a patient's submedian lesions (replicating the original trial primary efficacy outcome, 0.196pu) 580 people (at 80% power) and 776 people (at 90% power) would be required.

**Table 4:** Sample size calculations for future remyelination trials

	Number needed for trial (overall, not per arm)							
	Pure GM lesions		Pure CGM lesions		Pure DGM lesions		Brainstem lesions	
	Power		Power		Power		Power	
	80%	90%	80%	90%	80%	90%	80%	90%
Treatment effect (pu MTR)								
1.3	56	74	66	88	36	46	22	28
1.4	48	64	58	76	30	40	20	26
1.5	44	56	50	66	28	36	18	22
1.6	38	50	44	58	24	32	16	20
1.7	34	44	40	52	22	28	14	18
1.8	30	40	36	46	20	26	14	16
Observed baseline‐adjusted treatment effect in CCMR‐One trial (pu MTR)^1^	1.474		0.923		2.000		1.799	
Number (%) placebo‐treated patients with lesion type in CCMR‐One trial	18 (72%)		14 (56%)		8 (32%)		14 (56%)	
Mean number of lesions per placebo‐treated patient in CCMR‐One trial	2		1.6		0.4		1	

Sample size calculations for future remyelination trials using whole‐lesion metrics (patient‐level).

^1^ These values slightly differ to the Adjusted bexarotene‐placebo differences presented in Table 1 because the former are only adjusted for baseline value while the latter are additionally adjusted for the four binary minimisation factors prespecified in the trial: age (≤40/>40 years), gender, trial centre/scanner (Cambridge/Edinburgh) and EDSS (≤4·0/>4·0 score).

## Discussion

We have shown, for the first time in humans, that the response of demyelinated brain lesions to a remyelinating therapy varies within a person with multiple sclerosis, but also within individual lesions. Specifically, bexarotene had a significantly greater treatment effect on whole GM lesions but not whole WM lesions. Statistically significant treatment effects were detectable in WM lesion voxels with the lowest baseline MTR, but were missed when looking at the same tissue using whole‐lesion or lesion‐segment (regions defined by baseline T1‐hypointensity and MTR) approaches. Voxel‐level analyses also uncovered gradients of treatment effect in both WM and CGM lesional voxels (effects not visible at the whole lesion‐level), suggesting that treatment effects were lowest near brain surfaces. Finally, larger treatment effects were seen in the rims and surrounding regions of GM lesions compared to inner cores. Bexarotene also reduced the latency of prolonged visual evoked potentials in the same cohort, congruent with the conventional interpretation that increases in MTR reflect remyelination in chronic lesions.

While histopathological studies have suggested that the potential for GM remyelination may be greater than WM,^7^, ^8^, ^9^ this has not been shown directly in people with multiple sclerosis before, and studies assessing potential remyelinating treatments have not distinguished GM from WM lesions.^23^, ^24^, ^25^ GM lesions are more extensive than WM lesions, particularly in progressive multiple sclerosis (22.6% of GM and 8.3% of WM in people with secondary progressive multiple sclerosis).^26^ However, GM is intrinsically less myelinated than WM, hence previous difficulties detecting GM (compared with WM) lesions both histopathologically and with MRI. Using conventional MRI methods, less than 5% of GM lesions are detected, although with sequences such as double inversion recovery this increases to nearly 10%. In contrast in WM ~70% of lesions are detected.^27^ Accordingly, we detected substantially more WM (*n* = 1613) than GM (*n* = 106) lesions. Despite this, we still found an overall treatment effect in whole GM but not whole WM lesions. For future remyelination trials, we recommend sequences specifically designed to detect GM lesions.

WM lesions with higher MTR values may already have remyelinated to their maximum capacity, limited by the remaining axonal scaffold and gliosis. This may explain why treatment effects decreased with increasing baseline MTR in WM lesional voxels. Conversely axonal loss in WM lesions will limit the capacity for remyelination and lesion T1‐hypointensity is thought to reflect this better than a reduced MTR^28^, ^29^ (although both T1‐hypointensity and MTR correlate with axonal counts and myelin density^16^, ^29^). However, treatment effects did not vary by voxel T1 intensity in any lesion type, suggesting that MTR rather than T1 intensity (measured with a non‐quantitative T1‐weighted rather than a quantitative T1 sequence) better assesses remyelination potential. However, this effect is small compared with the differing effects seen in GM versus WM lesions. In CGM lesional voxels the opposite was seen: treatment effects increased with increasing baseline MTR. The cause of this is unclear, highlighting the need to better understand how different cellular substrates effect MTR in WM and GM lesions. For example, while the strongest correlate of MTR in WM lesions and CGM lesions is myelin density, astrocyte density (which may effect remyelination^30^) and macrophage density contribute significantly less to CGM lesion MTR than they do to WM lesion MTR.^19^

WM lesions close to the ventricles have a lower MTR,^12^, ^13^ a greater chance of becoming T1‐hypointense^11^ and longer T1 relaxation times than deep WM lesions (consistent with less remyelination).^14^ While proximity to brain surfaces did not influence treatment effects in whole WM nor GM lesions, voxel‐level analyses revealed significantly smaller treatment effects in WM lesional voxels near the ventricles which grew with increasing periventricular distance; and significantly smaller treatment effects in the outermost CGM lesion voxels (compared to the innermost voxels). In GM, “surface‐in” gradients of demyelination, neuronal and astrocyte loss are seen, and conversely greater glia limitans and microglial activation is seen closer to the surface of the brain,^31^, ^32^ though the pathological substrate of the MTR gradient in WM remains unknown. The surface‐in gradients of remyelination failure shown here may be driven by the same process that underlies cortical and periventricular gradients in demyelination and neuro‐axonal loss (with most evidence invoking a CSF‐mediated process secondary to meningeal inflammation^33^).

A significant number of WM lesions remain chronically active in their periphery rather than their core,^14^ where remyelination is known to be more extensive.^34^, ^35^ Given that we did not see an effect in whole WM lesions overall, it is perhaps unsurprising that we did not see a major difference in the core compared with the periphery of the same lesions. However, in GM lesions we additionally saw a greater treatment effect in lesion rims compared with their cores, consistent with the histopathological finding of greater remyelination in the periphery of GM lesions.^7^

Voxel‐level analyses identified statistically significant treatment effects which were missed with whole‐lesion or lesion‐segment (based on MTR and T1‐hypointensity) level analyses. This speaks to the heterogeneity of pathological change within lesions, including that repeated bouts of demyelination and (partial) remyelination may occur anywhere within a single lesion.^36^ To identify treatment effects within WM lesions, we recommend voxel‐level approaches in voxels with the lowest baseline MTR (though overall treatment effects were greater and considerably easier to measure in GM lesions).

We estimated sample sizes for trials based on patient‐averaged MTR measures in GM and brainstem lesions, and, based on the effect we observed, our study cohort was on the border of being sufficient to detect a treatment effect (80% power), and increasing the effect size from 1.3 pu to 1.8 pu (still well within a biologically plausible range for remyelination) reduces the total cohort size needed from 56 to 30 people. However, the optimal outcome measure requires consideration of multiple factors: the magnitude of the treatment difference a trial is being powered to detect must be both achievable and biologically meaningful (Table 4 representing 43%–60% of the maximum possible treatment effects suggested by MRI and ex‐vivo data)^20^; the outcome measure must be measurable in all participants (for example requiring GM lesions as an inclusion criterion) to avoid some participants not contributing to the outcome; and finally that the sample size is practical. Similarly, it is worth noting that this trial was 6 months long, and we do not know if bexarotene had had sufficient time for its maximal effect to be observed (VEPs continued to improve 28 months after stopping bexarotene).^37^ After an acute inflammatory episode remyelination continues for at least a year,^38^ so sensitivity to treatment effects may be meaningfully enhanced by extending trials by 3–6 months (though bexarotene's safety profile would have likely precluded this).

Some other limitations are worth noting. First is the size of the study itself. It was powered to detect a 1.16 pu patient‐averaged treatment effect across all lesions with a sub‐median baseline MTR, but we observed a baseline‐adjusted effect of 0.196 pu in such submedian lesions and a 1.474 pu effect in pure GM lesions. To determine if the increase in MTR in whole submedian WM lesions we observed is significant at 80% power, we would have needed a total sample of 580 people, but only 44 people to show an effect in pure GM lesions (and even smaller samples if using DGM or brainstem lesions). Second, as already noted, is the specificity of MTR for myelin. As axonal regeneration is essentially not seen in the adult central nervous system, increases in MTR in chronic lesions can reasonably be interpreted as representing remyelination. However, other MRI techniques such as myelin water fraction and quantitative magnetisation transfer measures may increase specificity for myelin.^39^, ^40^ Third, FLAIR detects less than 5% of GM lesions found with histopathology,^27^ so those detected here may not be generalisable to all GM lesions. Fourth, corroboration in different remyelination trial datasets is needed to examine whether different mechanisms demonstrate such marked changes in GM lesions. Fifth, non‐isotropic T2 and FLAIR were used for lesion identification and contouring, increasing partial volume effects at lesion surfaces (though this would affect both the bexarotene and placebo arms equally), so could not account for between‐arm treatment effects. We minimised this by excluding the outermost WM lesion voxels yet still found biologically plausible results. Sixth, although GM lesions appear more sensitive, they were not detected in 28% of patients under study and, when present, each patient contributed small numbers of GM lesions (Table 4). If GM lesions are to be used as a primary outcome measure, MRI sequences more sensitive to them, and the presence of GM lesions as an inclusion requirement, will be necessary. Seventh, sequences to reliably distinguish paramagnetic rim lesions were not collected, precluding subanalysis of within‐lesion spatial components in chronic active lesions. Eighth we cannot be certain that the same tissue was captured within a voxel between the baseline and follow‐up scans, though minimised this by using ~1 mm isotropic T1‐weighted and MTR scans in halfway space, and we found high correlation between baseline and follow‐up MTR intensity within individual lesional voxels (Supplementary Material), suggesting this was unlikely to have substantially influenced our results. Ninth, some analyses contained a statistically significant interaction term but no statistically‐significant treatment effect in any quartile or band being examined (e.g. within WM lesional voxels from any band defined by distance to the CSF, Fig. 2). This suggests that the underlying factor (in this case distance to CSF) *does* influence treatment effects on remyelination, though no significant effect is seen individually within any band or quartile. Finally, we did not adjust for multiple comparisons since we are investigating a number of different hypotheses, and in such contexts correction can be inappropriate.^41^, ^42^ We explore these issues further in the Supplementary Materials. Nevertheless, there is a danger of spurious significant results, and *p*‐values close to 0.05 should be interpreted sensibly with caution, and regarded as hypothesis‐generating, for examination in future studies.

In conclusion, this study confirms that different lesions have different capacities for remyelination in response to bexarotene. Of the features assessed, GM lesions showed the greatest potential to remyelinate. Our results also show that in early phase remyelination trials, assessing lesions and voxels with greater potential to demonstrate remyelination, rather than many lesions with a lower collective potential for repair, significantly increases sensitivity.

## Author Contributions

Conception and design of the study (JWLB, FP, DRA, NGC, JLJ, PC, SC, RF, RS, AC, DC), acquisition and analysis of data (JWLB, FP, DRA, NGC, JS, ZGG, EJN, CGW‐K, DM, JLJ, PC, SC, RF, RS, AC, DC), drafting a significant portion of the manuscript or figures (JWLB, FP, DRA, BK, NGC, CD, AC, DC).

## Conflict of Interest

William Brown reports consultancy fees, advisory board work and speaking fees from Novartis, Biogen and Intesso. Robin Franklin reports personal fees from Frequency Therapeutics and Rewind Therapeutics, outside the submitted work. Siddharthan Chandran reports funding from Phenotherapeutics, outside the submitted work. Declan Chard is a consultant for Hoffmann‐La Roche. In the last 3 years he has been a consultant for Biogen, has received research funding from Hoffmann‐La Roche, the International Progressive MS Alliance, the MS Society, the Medical Research Council, and the National Institute for Health Research (NIHR) University College London Hospitals (UCLH) Biomedical Research Centre, and a speaker's honorarium from Novartis. He co‐supervises a clinical fellowship at the National Hospital for Neurology and Neurosurgery, London, which is supported by Merck.

All other authors declare no competing interests of relevance to the current work.

## Supporting information


**Data S1.** Image Processing Pipeline, detailing the steps in lesion segmentation and tissue segmentation.
**Figure S1.** Axial slice from 3D‐weighted T1 image illustrating (A) the 10 white matter and deep grey matter (WMDGM) bands (with bands 1 and 10 excluded from analyses to mitigate partial volume effects); and (B) the 2 cortical grey matter (CGM) bands. Lesions are highlighted in green.
**Table S1.** Pearson correlation of lesional voxel MTR between baseline and follow‐up.
A note on correction for multiple comparisons.
