# Supplementary material for: Remyelination varies between and within lesions in multiple sclerosis following bexarotene
Source: Ann Clin Transl Neurol. 2022 Sep 17;9(10):1626–42. doi: 10.1002/acn3.51662 (PMC9539389; doi:10.1002/acn3.51662)
Supplement: Supplementary file 1 — Data S1. Image Processing Pipeline, detailing the steps in lesion segmentation and tissue segmentation. Figure S1. Axial slice from 3D‐weighted T1 image illustrating (A) the 10 white matter and deep grey matter (WMDGM) bands (with bands 1 and 10 excluded from analyses to mitigate partial volume effects); and (B) the 2 cortical grey matter (CGM) bands. Lesions are highlighted in green. Table S1. Pearson correlation of lesional voxel MTR between baseline and follow‐up. A note on correction for multiple comparisons. [file ACN3-9-1626-s001.docx]

**Supplementary Material**

**Image Processing**

Lesion segmentation

At month 0 lesions were identified on T2 sequences with the help of FLAIR using JIM v 6.0 (Xinapse Systems, Aldwincle, UK). They were identified by a blinded rater, contoured by a different blinded rater, and checked by a further blinded rater. The month 0 and month 6 volumetric T1 scans were registered to their halfway volumetric T1 space. The month 0 PD/T2 scans (plus the corresponding lesion mask) and the month 6 PD/T2 scans were affine co-registered to the month 0 and month 6 volumetric T1 scans respectively, using NiftyReg,^1^ then transformed to halfway volumetric T1 space using the transformations generated from the previous step. In this space, the halfway month 0 lesion mask was copied, laid over the month 6 PD/T2 scan, and any expanding or new lesions were manually adjusted to create a month 6 lesion mask of existing, expanding and new lesions. This approach enables the same tissue to be examined but means that subtle shrinkage or expansion of lesions cannot be captured.

Tissue segmentation

T1 volumetric scans in the halfway volumetric space were lesion filled using a patch-based method^2^ and then segmented into WM, DGM, CGM and cerebrospinal fluid (CSF) using the geodesic information flows.^3^ These segmentations were used for four purposes. First, to calculate brain parenchymal fraction (BPF): (GM + WM)/(GM + WM + CSF), reported previously.^4^ Second, to segment the WM and deep GM into 10 concentric bands and the cortex into 2 concentric bands (both performed using the normalised distance map derived from the normal to the Laplace equation isolines as previously described^5^; for normal-appearing (NA)WM and DGM, band 1 (nearest the inner ependymal surface) and band 10 (nearest the cortex) were excluded to minimise partial volume effects leaving 8 bands. These bands were used for lesion location (defined by the band containing the largest portion of a lesion) and voxel location, relative to the surface of the brain. Third to identify lesion and voxel location using the following definitions: (i) CGM lesion (a lesion entirely within the CGM segmentation); (ii) leukocortical (a lesion with voxels in both CGM and WM); (iii) juxtacortical (a lesion that is all WM and is mainly in the two WM bands closest to CGM); (iv) periventricular WM (a lesion that is all WM and the biggest proportion is within the four bands nearest the ventricles); (v) deep WM lesion (a lesion that is all WM and the biggest proportion is neither juxtacortical or periventricular); (vi) DGM (a lesion entirely within deep GM); (vii) mixed deep GM / WM (any lesion containing voxels within DGM and deep WM). Fourth, for an alternative lesion classification dividing lesions into those entirely within CGM or DGM (pure GM lesions), those entirely within WM (pure WM lesions) and those with mixed WM and GM parts (mixed GM / WM lesions). Classification methods were checked manually in each participant and confirmed to be accurate.


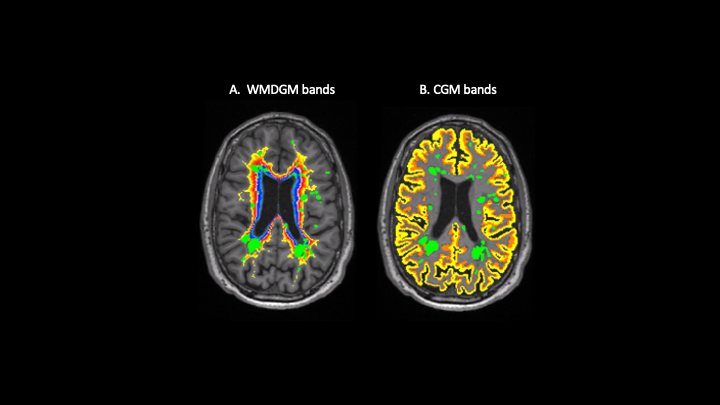
**Illustration of bands**

Figure S1: Axial slice from 3D-weighted T1 image illustrating (A) the 10 white matter and deep grey matter (WMDGM) bands (with bands 1 and 10 excluded from analyses to mitigate partial volume effects); and (B) the 2 cortical grey matter (CGM) bands. Lesions are highlighted in green.

**Pearson correlation of lesional voxel MTR between baseline and follow-up.**

|  | **Pure WM lesion voxels** | **Pure CGM lesion voxels** | **Pure DGM lesion voxels** |
| --- | --- | --- | --- |
| Pearson correlation coefficient between baseline & follow-up voxel MTR (95% confidence interval), p-value. | 0.955 (0.953-0.957)  *p* < 2.2x10^-16^ | 0.737 (0.710-0.764)  *p* < 2.2x10^-16^ | 0.789 (0.744 – 0.826)  *p* < 2.2x10^-16^ |

**A note on correction for multiple comparisons (by Dan Altmann, medical statistician)**

Correction for multiple comparisons should not be made simply because a large number of tests are reported: in order to appreciate why multiple comparison should be a considered, and to avoid a reflex reaction to multiple tests, we must recall that 5% of all tests whose null hypotheses are true are spuriously significant at 5%, with Type 1 error, regardless of whether they are reported in one or many published papers; we do not correct for multiple comparisons in one paper simply because we have published many papers in the past. Correction for multiple comparisons is appropriate in the following three main types of situation:

a) When there is a single null hypothesis which is tested using a number of comparisons, and where the significance of any one of these comparisons is sufficient to reject the single hypothesis; this situation is equivalent to having a joint null hypothesis, that all individual null hypotheses are jointly true, and where this joint null hypothesis is rejected if any individual hypothesis is rejected; an example may be where a trial treatment is to be considered effective if any one of a number of tested outcomes is significant.  In such a situation the Type 1 error on the single, joint hypothesis becomes much higher than the individual test Type 1 errors.

b) When the multiple tests are individually reported or highlighted not because of their relevance to prior individual hypotheses of interest, but because of being statistically significant; an example is where many imaging voxels are simultaneously tested for differences between two groups of patients, and only the significant voxels are highlighted.  Even though we would expect no more than 5% of the voxels overall to be significant at 5% if there were truly no difference between the groups, we would, if there were truly no difference, expect 100% of the highlighted voxels to be show Type 1 error – a false discovery rate of 100% - and so there would in general be a greatly increased chance of Type 1 error in this type of situation when testing hypotheses whose truth is unknown, where there is potential for a high proportion of true null hypotheses.  This is a kind of “dredging” or “fishing”, since there is no prior hypothesis about which regions will “light up” a significant. A similar situation occurs if a number of tests are done with no prior hypothesis and only the significant tests are reported in a paper simply because these are significant, without regard to prior hypotheses: 100% of these would be spurious, Type I errors, if there were truly no associations. By carefully focusing on plausible prior hypotheses, rather than testing “at random”, the false discovery rate can be enormously reduced.

c) When Type 1 error has much graver consequences than Type 2 error. For example, in the first of multiple interim trial analyses, the consequences of stopping early because the treatment effect is significant are much graver than the consequences of failing to detect a significant treatment effect at that stage. In other contexts Type 2 error may have worse consequences than Type 1 error, and it would be potentially damaging to correct for multiple comparisons at the expense of failing to detect a true result: for example when conducting a number of tests on the safety of a drug: the consequences of failing to detect a true harmful drug effect (Type 2 error) may be graver than falsely judging a drug to be harmful (Type 1 error).

None of these three situations applies in our case: we are not examining a single null hypotheses, but several; we are reporting significant and non-significant results, and highlighting the former in relation to prior and plausible hypotheses, not because of a fishing expedition for significant results; and, in the context of scientifically exploratory, hypothesis-generating context, the consequences of Type 2 error can be at least as important as Type 1 error.

Finally, the multiple comparison issue assumes a binary interpretation of p-values: statistically significant yes vs no. In truth, p-values are a continuous measure of the evidence against a null hypothesis and in support of the alternative hypothesis: as such, they do not, cannot, and must not be considered as a method of deciding truth, but need to be considered carefully and in context, particularly regarding the plausibility of hypotheses and possible underlying mechanisms. If this is properly considered, the motivation and concern for multiple comparison, in contexts which are not one of the three outlined above, is much reduced. These and other related issues are discussed further in these references.^6,7^

**References**

1. Modat M, Cash DM, Daga P, Winston GP, Duncan JS, Ourselin S. Global image registration using a symmetric block-matching approach. *J Med Imaging (Bellingham)*. Jul 2014;1(2):024003. doi:10.1117/1.JMI.1.2.024003

2. Prados F, Cardoso MJ, Kanber B, et al. A multi-time-point modality-agnostic patch-based method for lesion filling in multiple sclerosis. *NeuroImage*. Jul 1 2016;139:376-384. doi:10.1016/j.neuroimage.2016.06.053

3. Cardoso MJ, Modat M, Wolz R, et al. Geodesic Information Flows: Spatially-Variant Graphs and Their Application to Segmentation and Fusion. *IEEE Trans Med Imaging*. Sep 2015;34(9):1976-88. doi:10.1109/TMI.2015.2418298

4. Brown JWL, Cunniffe NG, Prados F, et al. Safety and efficacy of bexarotene in patients with relapsing-remitting multiple sclerosis (CCMR One): a randomised, double-blind, placebo-controlled, parallel-group, phase 2a study. *The Lancet Neurology*. Sep 2021;20(9):709-720. doi:10.1016/S1474-4422(21)00179-4

5. Pardini M, Sudre CH, Prados F, et al. Relationship of grey and white matter abnormalities with distance from the surface of the brain in multiple sclerosis. *Journal of neurology, neurosurgery, and psychiatry*. Sep 6 2016;doi:10.1136/jnnp-2016-313979

6. Perneger TV. What's wrong with Bonferroni adjustments. *BMJ*. Apr 18 1998;316(7139):1236-8. doi:10.1136/bmj.316.7139.1236

7. Rothman KJ. No adjustments are needed for multiple comparisons. *Epidemiology*. Jan 1990;1(1):43-6.
